# Constructing safe and durable antibacterial textile surfaces using a robust graft-to strategy via covalent bond formation

**DOI:** 10.1038/srep36327

**Published:** 2016-11-03

**Authors:** Liang He, Sha Li, Cordelia T. W. Chung, Chang Gao, John H. Xin

**Affiliations:** 1 Institute of Textiles and Clothing, The Hong Kong Polytechnic University, Hong Kong, China

## Abstract

Recently zwitterionic materials have been widely applied in the biomedical and bioengineering fields due to their excellent biocompatibility. Inspired by these, this study presents a graft-to strategy via covalent bond formation to fabricate safe and durable antibacterial textile surfaces. A novel zwitterionic sulfobetaine containing triazine reactive group was specifically designed and synthesized. MTT assay showed that it had no obvious cytotoxicity to human skin HaCaT cells as verified by ca. 89.9% relative viability at a rather high concentration of 0.8 mg·mL^−1^. In the evaluation for its skin sensitization, the maximum score for symptoms of erythema and edema in all tests were 0 in all observation periods. The sulfobetaine had a hydrophilic nature and the hydrophilicity of the textiles was enhanced by 43.9% when it was covalently grafted onto the textiles. Moreover, the textiles grafted with the reactive sulfobetaine exhibited durable antibacterial activities, which was verified by the fact that they showed antibacterial rates of 97.4% against gram-positive *S. aureus* and 93.2% against gram-negative *E. coli* even after they were laundered for 30 times. Therefore, the titled zwitterionic sulfobetaine is safe to human for healthcare and wound dressing and shows a promising prospect on antibacterial textile application.

Bio-fouling is an important issue in many biological applications because non-specific adsorption of micro-organisms will compromise the target performances of medical devices^1^. It has always been a main topic to develop antifouling surfaces on these medical devices to reduce non-specific adsorptions. Bio-inspired by the inert nature of cell plasma membranes, zwitterionic phosphorylcholines were used to fabricate low fouling surfaces^2^,^3^,^4^. Because phosphorylcholines were difficult to synthesize, zwitterionic sulfobetaines and carboxybetaines were developed to fabricate antifouling surfaces on various materials due to their easy preparation and excellent non-fouling properties^5^. Currently, these zwitterionic betaines are being widely used in a variety of biological and medical applications including surface coatings, drug/gene carriers and antibacterial materials^1^,^6^,^7^,^8^,^9^. Although the exact mechanism describing the non-fouling property has not yet been completely understood, it is generally considered that the strong hydration ability through ionic solvation of these materials plays a key role^10^,^11^. For example, it was showed that one sulfobetaine structure could tightly bind with 7~8 water molecules, achieving in excellent hydrophilicity^12^. From the perspective of molecular structure, sulfobetaine is very close to taurine (2-aminoethane sulfonic acid), which abundantly presents in tissue cells of human and animals^13^. Thus, sulfobetaine shows good biocompatibility. Due to these advantages, sulfobetaines are very promising as excellent candidates for the surface functionalization on textile materials. They could not only provide antibacterial surfaces on textile materials for healthcare and wound dressing, but also enhance the hydrophilicity of the textiles, which is favorable to the skin moisturizing of the wounds. However, very few sulfobetaine derivatives have been reported for the functionalization on textile materials^14^.

The methods for fabricating zwitterionic surfaces are generally classified into two approaches of graft-from and graft-to^5^,^10^. In the “graft-from” method, the target zwitterionic layers are usually to be synthesized directly on the substrate surfaces via the steps of initiator immobilization on the surfaces and initiator initiated polymerization^15^. Atom transfer radical polymerization (ATRP) is a typical and popular graft-from method to achieve the zwitterionic surfaces with expected surface functional performances^15^,^16^,^17^. The grafting densities and layer components are able to be controlled based on the purpose. However, the graft-from method requires harsh conditions including the grafted initiators and oxygen-free atmosphere. Metal catalysts are also required in this method, so it cannot be used in certain systems, where metal contents are strictly limited. In the graft-to method, the zwitterionic derivatives are usually pre-synthesized in advance and then grafted onto the target substrate surfaces through chemical anchoring reactions^15^,^18^. In this case, these zwitterionic derivatives often contain adhesive catechol, thiol, silane or hydroxyl groups acting as anchors to immobilize them on substrate surfaces^18^,^19^,^20^. In comparison with graft-from method, the graft-to method is more convenient and widely used to fabricate zwitterionic derivatives onto substrate surfaces^15^,^21^,^22^,^23^. But, using these chemical anchors in the graft-to method is hard to achieve the high grafting densities of zwitterionic derivatives on substrate surfaces. And their grafted stabilities are often limited, leading to a compromised performance in the application^23^,^24^. In practical applications, it is always desirable and preferred to use a more convenient and easier method to graft zwitterionic derivatives stably onto substrate surfaces with high grafting densities. Therefore, a more powerful method for the grafting of zwitterionic surfaces is still needed to meet these application requirements.

Cellulosic textiles are the largest amounts of textile materials consumed by human and they almost full-time protect our skin closely. For healthcare concern, it is worthy to study the antibacterial applications of zwitterionic materials on textiles because of their excellent biocompatibility and specific surface properties. In order to obtain the antibacterial benefits of zwitterionic surfaces with high grafting densities on cellulosic textiles, a robust graft-to strategy via stable covalent bond formation is proposed herein based on the hydroxyl-enriched nature of cellulosic textiles. Accordingly, a novel kind of reactive zwitterionic sulfobetaine (PSBC) was designed and synthesized (Fig. 1). Due to the high reactivity, low cost and easy availability, cyanuric chloride is an ideal candidate as a reactive group for the proposed grafting via covalent bond formation^25^. The covalent grafting was carefully characterized and its grafting stability was investigated by the evaluation of the antibacterial durability of the grated textiles. The acute cytotoxicity, skin irritation and hydrophilicity of PSBC were also studied based on the requirements of textile applications.

## Results

### The covalent grafting mechanism

**Figure 2:**
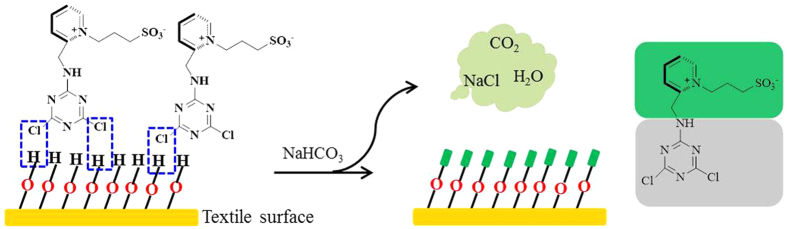
A scheme of the fabrication process. The graft-to mechanism via covalent bond formation between the textiles and PSBC.

The covalent grafting of PSBC onto the textiles was achieved in a typical padding-curing process. In this procedure, PSBC together with alkaline agent was first padded onto the textiles through the adsorption at room temperature. The padded textiles were undergone a curing in an oven, where the etherification took place to form permanent covalent bonds between the reactive triazine group in PSBC and hydroxyl groups on the textile surfaces, as shown in Fig. 2. Due to the good solubility in water, the un-grafted PSBC on textile surfaces were easily washed away during the washing step. Therefore, the zwitterionic textile surfaces were fabricated using PSBC through the robust graft-to approach via covalent bond formation.

### Characterizations of the grafted textiles

**Figure 3:**
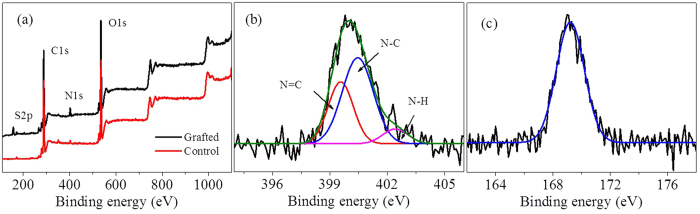
XPS analysis of the textiles. (**a**) Survey spectra for raw textiles and the textiles grafted with PSBC. (**b**) The N1s high resolution spectra of the textiles grafted with PSBC. (**c**) The S2p spectra of the textiles grafted with PSBC.

The textiles grafted with PSBC were first characterized using XPS analysis to determine their surface elemental compositions. Figure 3 was shown their survey scan spectra and high resolution spectra. In Fig. 3a, the raw textiles had two strong peaks at ca. 285.8 and 533 eV attributable to C1s and O1s, respectively^26^. Except these two peaks, the textiles grafted with PSBC clearly showed another two peaks at ca. 400.3 and 169.2 eV and they were attributed to N1s and S2p^14^,^26^, both of which certainly originated from the PSBC grafted on the textile surfaces. In Fig. 3b, the N1s high resolution spectra of the textiles grated with PSBC could be curve-fitted three peak components at the binding energy of 399.6, 400.5 and 402.4 eV, which are attributed to N = C, N-C and N-H species, respectively^27^. According to their integral areas of N species, the molar ratio of N-C, N = C and N-H was calculated as 4.16:7.18:1.0, which was close to the theoretical ratio of 4:7:1 in PSBC. The calculated ratio of SO_3_^−^ (based on integral area of S2p, Fig. 3c) to N^+^ (anion to cation) for the textiles grafted with PSBC was 1.38 according to the molar percentage of N = C specie, which was similar to those calculated from the molar percentages of N-C or N-H species. The obtained ratio of SO_3_^−^ was higher than that of N^+^. which indicated that the sulfobetaine groups were enriched on the textile surfaces^14^,^28^, and the anionic heads of PSBC stretched apart from the negatively charged textile surfaces due to the charge repulsions.

**Figure 4:**
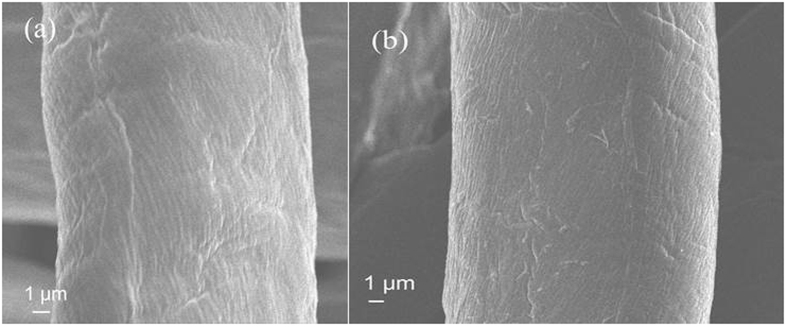
Micro-morphology of the textile fibers. (**a**) SEM image of raw textiles. (**b**) SEM image of the textiles grafted with PSBC. Magnification: ×3500.

The surface morphology of the raw textiles and the textiles grafted with PSBC were investigated using SEM analysis. As shown in Fig. 4a, many wrinkles were clearly observed on the surfaces of the raw textiles. After the covalent grafting, the textiles still showed rich regular wrinkles on their surfaces (Fig. 4b). Furthermore, the textile surfaces were not destroyed under the grafting conditions, showing the feasibility of the grafting procedure. There were no obvious film layers and particles attachments on the grafted textile surfaces. From these SEM analyses, it was indicated that there were no obvious changes in the surface morphology of the textiles after the covalent grafting, which was advantageous to retain their original characteristics, such as air permeability.

### Physical performance of the grafted textiles

**Figure 5:**
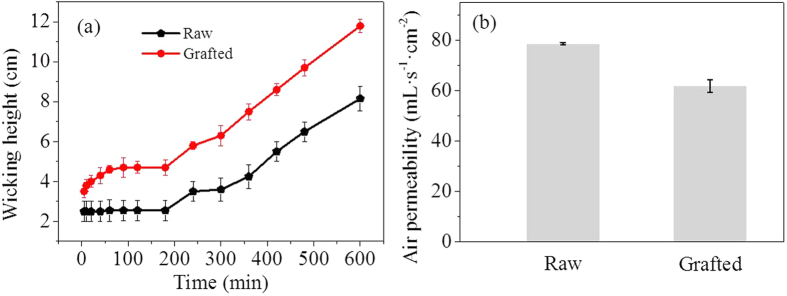
The physical performances of raw textiles and the textiles grafted with PSBC. (**a**) The comparison of their hydrophilicity. (**b**) The comparison of their air permeability. Average of three tests was used.

Figure 5a showed the hydrophilic results of the raw textiles and the textiles grafted with PSBC by the measurements of their wicking heights. The water wicking heights of the raw textiles had no obvious changes after initial 5 min and were then slowly increased until 180 min. However, the water wicking heights of the textiles grafted with PSBC gradually increased from the beginning, showing different hydrophilic properties as compared to raw textiles. Within 120 min, the raw textiles had the water wicking heights of 2.6 cm, while the textiles grafted with PSBC had the water wicking heights of 4.7 cm. Until 420 min, the water wicking heights of the raw textiles increased to 5.5 cm, while the heights of the textiles grafted with PSBC increased to 8.6 cm. In successive wicking to 600 min, the raw textile had the water wicking heights of 8.2 cm. On the contrary, the textiles grafted with PSBC had the water wicking heights of 11.8 cm in this stage. According to the wicking heights within 600 min, the hydrophilicity of the raw textiles was obviously enhanced by 43.9% after the covalent grafting with PSBC. These hydrophilic results indicated their different surface properties induced by PSBC, which further confirmed its successful grafting onto the textile surfaces.

Air permeability is an important property to textile materials because it usually has great influences on wound recovery in the application. PSBC has the nature of small molecules, so the original surface morphology of the raw textiles was not destroyed after the covalent grafting with PSBC, as verified by their SEM images (Fig. 4). Thus, the air permeability of the textiles was affected only by the grafted PSBC molecular layer. After the grafting, the air permeability of the textiles reduced from ca. 78.7 to 61.9 mL·s^−1^·cm^−2^ (Fig. 5b), showing a reduction by 21.3%. This reduction was relative mild compared to the reduction from those coatings of polymer and nanoparticles^29^,^30^.

### Antibacterial durability of the textiles grafted with PSBC

**Figure 6:**
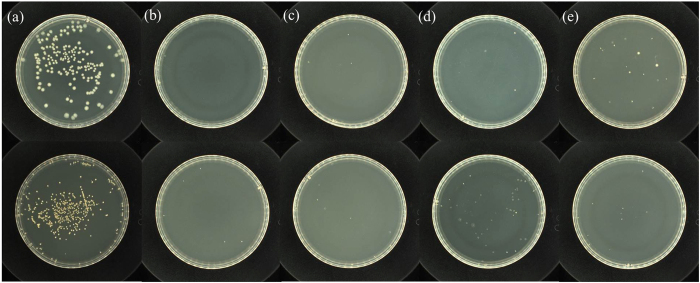
The bacteria reproductions of gram-negative *E. coli* (up) and gram-positive *S. aureus* (bottom) on the textiles. (**a**) Raw textiles. (**b**) The grafted textiles by PSBC without laundering. (**c**) The grafted textiles by PSBC being laundered for 10 times. (**d**) The grafted textiles by PSBC being laundered for 20 times. (**e**) The grafted textiles by PSBC being laundered for 30 times.

In compared with the raw textiles, the textiles grafted with PSBC had been laundered dozens of times for the evaluation of antibacterial durability, as shown in Fig. 6. For the raw textiles, both gram-negative *E. coli* and gram-positive *S. aureus* bacteria colonies reproduced on the most parts of the dishes after 24 h culture. By contrast, nearly no bacteria colonies reproduced on the dishes for the textiles grafted with PSBC, which showed the superior antibacterial activity of the grafted PSBC surface layers. Until the grafted textiles had been laundered for 10 times, several bacteria colonies’ reproduction of *E. coli* and *S. aureus* was observed on the dishes, which indicated that a very tiny fraction of the grafted PSBC left away from the textile surfaces during the laundering process. When the grafted textiles had been further laundered for 20 and 30 times, there were no much changes in the bacteria colonies’ reproduction of *E. coli* and *S. aureus*. This suggested that no more grafted PSBC left away from the textile surfaces and the titled grafting via covalent bonds formation on textiles was stable to the repeated launderings.

**Figure 7:**
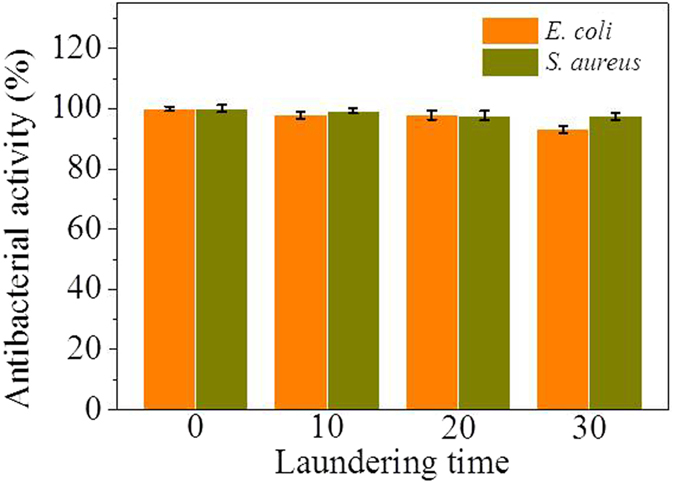
The antibacterial activities. The antibacterial rates of the textiles grafted by PSBC against gram-negative *E. coli* and gram-positive *S. aureus* versus laundering time. Average of three tests was used.

Based on the results of the bacteria colony reproduction on the textiles (Fig. 6), their antibacterial rates were calculated for the evaluation of antibacterial activity versus laundering time. The results were shown in Fig. 7. The grafted textiles had the antibacterial rates over 99.9% against both gram-negative *E. coli* and gram-positive *S. aureus* as compared with the raw textiles. Until laundered for 20 times, the grafted textiles showed only slight reductions in the antibacterial rates by ca. 2.0%. After laundered for 30 times, the grafted textiles still had antibacterial rates of 97.4% for *S. aureus* and 93.2% for *E. coli*, which showed a slight higher antibacterial activity to gram-positive *S. aureus* than to gram-negative *E. coli*. All these results of antibacterial rates indicated that the textiles grafted with PSBC exhibited durable antibacterial activities against both gram-positive *S. aureus* and gram-negative *E. coli* bacteria in the repeated launderings, which further confirmed the grafting stability of PSBC onto the textiles through the covalent bond formation.

### Evaluation of application safety

**Figure 8:**
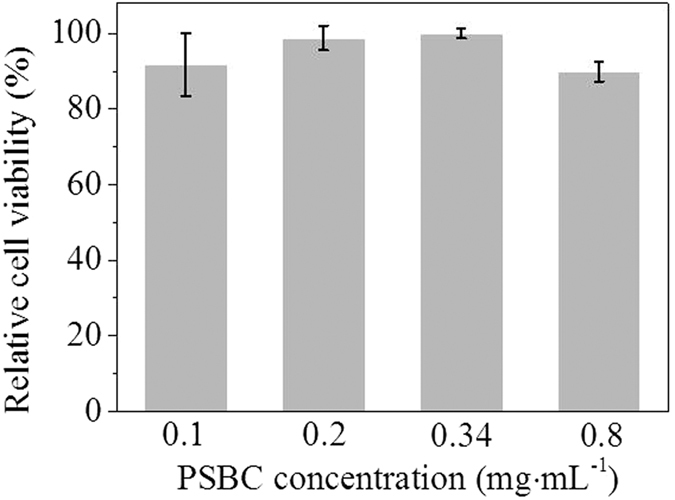
Cytotoxicity evaluation. The relative HaCaT cell viabilities after incubating with different concentrations of PSBC solution. Average of three tests was used.

In order to evaluate the application safety of PSBC on textiles, the cytotoxicity and skin irritation of PSBC were further evaluated. The cytotoxicity of PSBC was tested through the incubation of HaCaT cells with PSBC solution via classic MTT assays. Figure 8 showed the relative viabilities of HaCaT cells at different PSBC concentrations. Under the concentration of 0.1 mg·mL^−1^ PSBC, the HaCaT cells had a relative viability of 91.7% after they were incubated for 48 h in the solution. When the PSBC concentrations were further increased, the relative viabilities of the HaCaT cells had no clear decreases. Even at a rather high concentration of 0.8 mg·mL^−1^, the relative viability of the HaCaT cells was still at a high level of 89.9%. These assay results indicated that the cytotoxicity of PSBC was very low to human skin HaCaT cells even at an extremely high concentration.

The skin irritation evaluation results of PSBC were listed in Table 1. It can be seen that all the scores for the symptoms of erythema and edema were 0 on all tested rabbits at each observation periods, although the maximum scores of the dermal symptoms was recorded in the tests. Namely, the maximum scores of erythema and edema on each test rabbits at all observation stage was 0, which meant that no symptoms of erythema and edema were observed on all of the tested rabbits. According to these results, the skin irritation intensity of PSBC was determined as no irritation in its application on textiles.

## Discussions

The reactive zwitterionic PSBC was synthesized through a four-step reaction under mild conditions and its structure was fully confirmed by the spectral analyses of HRMS, ^1^H NMR and ^13^C NMR. In the fields of dyestuff chemistry, triazine is one of the widely used reactive groups for the reaction with cellulosic fibers^25^. It shows high reactivity and easily forms covalent bonds with cellulosic hydroxyl groups under mild conditions, thus was fixed on textile fibers in a non-leaching way. With these advantages in mind, in this study triazine was designed to be as an anchor to graft antibacterial betaine groups onto textile fibers permanently to endow textiles a durable antibacterial activity. This feasibility was successfully confirmed by their antibacterial measurements against launderings. Compared with the raw textiles, the grafted textiles showed over 99.9% antibacterial rates against both gram-negative *E. coli* and gram-positive *S. aureus* before laundering. After 30 times of launderings, they still exhibited antibacterial rates of 97.4% and 93.2% against *S. aureus* and *E. coli*, respectively, showing an excellent antibacterial durability. Obviously, it provided a robust strategy to easily fabricate non-leaching antibacterial textile surfaces. PSBC has the small molecular nature, and no particle attachments or polymer films existed on textile surfaces after its grafting, which was completely different from those nanoparticle and macromolecule coatings^30^. Thus, the porosity of textile fibers was not destroyed and the grafted textiles had a better air permeability compared to those nanoparticle and macromolecule coatings^29^,^30^. PSBC held the good hydrophilic character of betaine and could enhance the hydrophilicity of the textiles by 43.9% after it was grafted on textile surfaces, which is favorable to improve the moisturizing ability of the grafted textiles. These characteristics of the grafted textiles by PSBC are advantageous to the healing and recovery of wounds when they are applied for healthcare and wound dressing purpose. In the MTT assay, the relative viability of the HaCaT cells incubated by PSBC was still up to 89.9% at a rather high concentration of 0.8 mg·mL^−1^ PSBC. At the same relative cell viability, this concentration was much higher than that of some nanoparticles^31^. Skin sensitization evaluation of PSBC was assessed on rabbits according to standard methods. In this method, the evaluation score <0.5 was defined as no irritation^14^. After 72 h observation period, the scores for the symptoms of erythema and edema were 0 on all tested rabbits, demonstrating non-irritant performances. These results exhibited the biocompatibility of PSBC in its application on textiles. Therefore, PSBC is a promising alternative to endow textile materials safe and durable antibacterial activities for healthcare and wound dressing applications.

## Methods

### Materials

1,3-Propanesultone (PrS), 2-(aminomethyl)pyridine (AmP), di-*t*-butyl dicarbonate (BOC) and cyanuric chloride (CyC) were purchased from Sigma-Aldrich and used as received. Gram-positive *S. aureus* (ATCC 6538) and gram-negative *E. Coli* (ATCC 25922) were purchased from Guangdong Institute of Microbiology. HaCaT cells were received from Kunming Cell Bank of Type Culture Collection, Chinese Academy of Sciences. All other regents and solvents were commercially analytical grade and spectroscopic grade, respectively.

### Characterizations

High resolution mass spectra (HRMS) were recorded on a Micromass Q-TOF 2 mass spectrometer. Nuclear magnetic resonance (NMR) spectra were recorded on a Varian 400 spectrometer using TMS as an internal standard. Field emission scanning electron microscopy (SEM) images were obtained using a JEOL JSM-6335F microscopy. X-ray photoelectron spectroscopy (XPS) was used to analyze the elemental compositions of materials using a SKL-12 X-ray photoelectron spectrometer (Shenyang, China) equipped with a VG CLAM 4MCD electron energy analyzer.

### Synthesis of PSBC

Typically, AmP (1.1 g, 10.2 mmol) was first dissolved in DCM (15 mL) at room temperature. Under stirring, BOC (2.3 g, 10.5 mmol) solution in DCM (5 mL) was added and the obtained solution was stirred for another 7 h. Then, it was transferred into a funnel and washed with saline water three times (250 mL). The organic layer was collected and vacuum-concentrated at 50 °C to obtain **2** as yellow oil. The obtained **2** and PrS (1.6 g, 13.1 mmol) were refluxed overnight in chloroform (25 mL). The solvent was then removed to obtain the crudes of **3** as a brown solid. It was then dissolved in acidic D. I. water (20 mL) with the pH < 1. The resultant solution was stirred for 3 h at room temperature and then was concentrated under vacuum to remove the water, affording **4** as a yellow solid. Next, **4** and potassium carbonate (1.6 g, 11.6 mmol) were stirred in acetone (100 mL) for 30 min in an ice bath. The acetone solution (20 mL) of CyC (2.2 g, 11.9 mmol) was added and the resultant solution was stirred for another 8 h in the ice bath. After filtration, washing with acetone and dryness, PSBC was obtained as yellow powder. HRMS (ESI positive): *m/z* = 378.0191 ([M^+^ H]^+^), Calcd. for C_12_H_14_Cl_2_N_5_O_3_S = 378.0189. ^1^H NMR (D_2_O/DMSO-*d*_*6*_, ppm): 8.82 (d, 1H), 8.40 (t, *J* = 8.0, 1H), 7.93 (q, *J* = 8.0, 2H), 4.97 (s, 2H), 4.73 (t, *J* = 8.0, 2H), 2.85 (t, 2H), 2.32 (t, *J* = 8.0, 2H). ^13^C NMR (D_2_O, ppm): 170.30, 169.64, 165.95, 160.27, 153.48, 146.19, 127.08, 56.16, 47.18, 41.54, 25.36.

### Covalent grafting of PSBC onto textiles

Before use, the commercially available cotton raw textiles were cleaned in 5‰ solution of sodium dodecyl sulfate and followed by the full rinsing in deionized water. A piece of dried textile (30 × 40 cm^2^) was padded at 3 kg pressure in aqueous solution containing 20 g·L^−1^ CSPB and 10 g·L^−1^ NaHCO_3_. The resultant textile was cured at 90 °C for 3 min. After rinsed in tap water, the textiles were dried in air for use.

### Hydrophilicity and air permeability measurements

The hydrophilicity of the textiles was measured according to the standard method ISO 9073–6:2000. Typically, a piece of sample with the size of 25 × 3.0 cm^2^ on a backbone was conditioned at 25 °C and 70% relative humidity. Then, it was vertically put into a beaker filled with red water and the sample bottom was 2 cm lower than the water surface, which was set as the zero point. Thereafter, the water wicking height on the sample was recorded. The higher water wicking height means the better hydrophilicity. Air permeability of the textiles was obtained according to the rate of air flow passing perpendicularly through the tested textiles. The samples with 5.0 cm^2^ were tested on M021S Tester under 100 kPa according to the standard test method ASTM D737. A blank sample with the same curing procedure was used as control. The average of three measurements was used in the study.

### Evaluation of antibacterial activity

The antibacterial activity of the grafted textiles with CSPB was quantitatively evaluated against the representative bacteria of gram-negative *E. coli* and gram-positive *S. aureus* using viable cell count method^32^. To investigate the grafting stability of PSBC on textiles, the antibacterial activity of the grafted textiles was evaluated against repeated launderings according to AATCC standard method 61:2A-2010. The laundered samples were fully rinsed in D. I. water and dried in air for the test. The antibacterial activity of the grafted textiles was determined according to AATCC standard method 100–2012. The bacteria colonies on the agar plate were counted and the reduction rate was calculated according to Eq. (1)^33^.


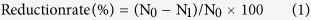


where N_0_ and N_1_ are the bacteria colonies after 24 h contact on the raw textiles and on the textiles grafted with CSPB, respectively. The average of three tests was reported.

### MTT assay

The HaCaT cells were seeded in 96-well plates and grew for 48 h in K-FSM medium with 10% fetal bovine serum at 37 °C and 5% CO_2_ before the treatment. The cells reached 80% confluency on the treatment day. Then, the medium was replaced with the fresh medium containing PSBC at various concentrations. After incubating 48 h, the medium was replaced with 100 μL thiazolyl blue tetrazolium bromide solution (0.5 mg·mL^−1^). 4 h later, the medium was replaced with 150 μL DMSO. Followed by shaking 10 min, the absorbance of the supernatants was measured at 492 nm using a microplate reader to calculate the cell viability. Cells without incubation with CSPB were used as control (100%). The cell viability was calculated according to Eq. (2)^34^.


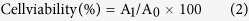


where A_0_ and A_1_ are the absorbance for the control and the samples incubated with PSBC, respectively. For each concentration, the average of three tests was reported.

### Evaluation of skin irritation

Skin irritation was evaluated according to the standard method in the biological evaluation of medical devices ISO 10993-10:2010(E) by SGS-CSTC Standards Technical Services Co., Ltd. Guangzhou Branch. In the experiment, it was used 3 healthy young adult New Zealand white albino rabbits (1 male and 2 female) with 2.2–2.4 kg, which were from Guangdong Medical Experiment Animal Center Sanshui Base (Certificate No. SCXK 2014-0035, Guangdong). All methods involving the animals were carried out in accordance with the approved guidelines for animal experiments of Guangdong Medical Experiment Animal Center Sanshui Base and were approved by the animal use committee of Guangdong Medical Experiment Animal Center Sanshui Base. The rabbit fur with the area of 10 × 15 cm^2^ was shaved before the test. The grafted textile with the size of 2.5 × 2.5 cm^2^ was applied on the test skin site with a gauze patch after it was moistened with 0.9% NaCl solution. Then, it was wrapped with nonirritating tape and bandage. The sample only moistened with 0.9% NaCl solution was as control. At each observation period, the dermal symptoms of erythema and edema at the tested sites were evaluated. The maximum score of the dermal symptoms was used in the evaluation. In evaluation criteria, score ranges of <0.5, 0.5 ~ 2.0, 2.0 ~ 6.0 and ≥ 6.0 are defined as no irritation, slight irritation, middle irritation and strong irritation, respectively.

## Additional Information

**How to cite this article**: He, L. *et al*. Constructing safe and durable antibacterial textile surfaces using a robust graft-to strategy via covalent bond formation. *Sci. Rep.* **6**, 36327; doi: 10.1038/srep36327 (2016).

**Publisher’s note:** Springer Nature remains neutral with regard to jurisdictional claims in published maps and institutional affiliations.

## Figures and Tables

**Figure 1:**
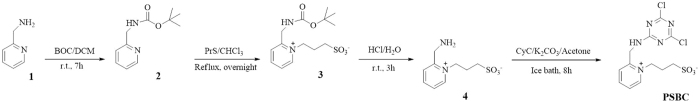
Synthesis process. Synthetic routes of PSBC using 2-(aminomethyl)pyridine as raw material.

**Table 1:** Skin irritation evaluation results of PBSC.

Time	Sample	Symptom	No. 1	No. 2	No. 3	Intensity of skin irritation
1 h	Finished	Erythema	0	0	0	
		Edema	0	0	0	
	Control	Erythema	0	0	0	
		Edema	0	0	0	
24 h	Finished	Erythema	0	0	0	
		Edema	0	0	0	
	Control	Erythema	0	0	0	
		Edema	0	0	0	No irritation
48 h	Finished	Erythema	0	0	0	
		Edema	0	0	0	
	Control	Erythema	0	0	0	
		Edema	0	0	0	
72 h	Finished	Erythema	0	0	0	
		Edema	0	0	0	
	Control	Erythema	0	0	0	
		Edema	0	0	0	

## References

[b1] Banerjee I., Pangule R. C. & Kane R. S. Antifouling coatings: recent developments in the design of surfaces that prevent fouling by proteins, bacteria, and marine organisms. Adv. Mater. 23, 690–718 (2011). 20886559 10.1002/adma.201001215

[b2] Chen S., Zheng J., Li L. & Jiang S. Strong resistance of phosphorylcholine self-assembled monolayers to protein adsorption: insights into nonfouling properties of zwitterionic materials. J. Am. Chem. Soc. 127, 14473–14478 (2005). 16218643 10.1021/ja054169u

[b3] Zhou W. . Zwitterionic phosphorylcholine as a better ligand for gold nanorods cell uptake and selective photothermal ablation of cancer cells. Chem. Commun. 46, 1479–1481 (2010). 10.1039/b915125g 20162154

[b4] Chen Y. . Zwitterionic supramolecular prodrug nanoparticles based on host-guest interactions for intracellular drug delivery. Polymer 97, 449–455 (2016).

[b5] Jiang S. & Cao Z. Ultralow-fouling, functionalizable, and hydrolyzable zwitterionic materials and their derivatives for biological applications. Adv. Mater. 22, 920–932 (2010). 20217815 10.1002/adma.200901407

[b6] Cao Z. . Reversibly switching the function of a surface between attacking and defending against bacteria. Angew. Chem. Int. Ed. 51, 2602–2605 (2012). 10.1002/anie.201106466 22213162

[b7] Mi L. & Jiang S. Integrated antimicrobial and nonfouling zwitterionic polymers. Angew. Chem. Int. Ed. 53, 1746–1754 (2014). 10.1002/anie.201304060 24446141

[b8] Jin Q., Chen Y., Wang Y. & Ji J. Zwitterionic drug nanocarriers: a biomimetic strategy for drug delivery. Colloids Surf. B 124, 80–86 (2014). 10.1016/j.colsurfb.2014.07.013 25092584

[b9] Unnithan A. R. . Electrospun zwitterionic nanofibers with *in situ* decelerated epithelialization property for non-adherent and easy removable wound dressing application, Chem. Eng. J. 287, 640–648 (2016).

[b10] He M. . Zwitterionic materials for antifouling membrane surface construction. Acta Biomater., 10.1016/j.actbio.2016.03.038 (2016). 27025359

[b11] Hower J. C. . Hydration of “nonfouling” functional groups. J. Phys. Chem. B 113, 197–201 (2009). 19072165 10.1021/jp8065713

[b12] Wu J., Lin W., Wang Z. & Chen S. Investigation of the hydration of nonfouling material poly(sulfobetaine methacrylate) by low-field nuclear magnetic resonance. Langmuir 28, 7436–7441 (2012). 22512533 10.1021/la300394c

[b13] Huxtable R. J. Physiological actions of taurine. Physiol. Rev. 72, 101–163 (1992). 1731369 10.1152/physrev.1992.72.1.101

[b14] Chen S. . Environmentally friendly antibacterial cotton textiles finished with siloxane sulfopropylbetaine. ACS Appl. Mater. Interfaces 3, 1154–1162 (2011). 21417413 10.1021/am101275d

[b15] Liu B. . Design and mechanisms of antifouling materials for surface plasmon resonance sensors. Acta Biomater. 10.1016/j.actbio.2016.02.035 (2016). 26921775

[b16] Sin M.-C., Sun Y.-M. & Chang Y. Zwitterionic-based stainless steel with well-defined polysulfobetaine brushes for general bioadhesive control. ACS Appl. Mater. Interfaces 6, 861–873 (2014). 24351074 10.1021/am4041256

[b17] Zhu Y. . Cellulose paper sensors modified with zwitterionic poly(carboxybetaine) for sensing and detection in complex media. Anal. Chem. 86, 2871–2875 (2014). 24571794 10.1021/ac500467c

[b18] García K. P. . Zwitterionic-coated “stealth” nanoparticles for biomedical applications: recent advances in countering biomolecular corona formation and uptake by the mononuclear phagocyte system. Small 10, 2516–2529 (2014). 24687857 10.1002/smll.201303540

[b19] Zhu Y. . A robust graft-to strategy to form multifunctional and stealth zwitterionic polymer-coated mesoporous silica nanoparticles. Biomacromolecules 15, 1845–1851 (2014). 24670217 10.1021/bm500209a

[b20] Nowinski A. K., White A. D., Keefe A. J. & Jiang S. Biologically inspired stealth peptide-capped gold nanoparticles. Langmuir 30, 1864–1870 (2014). 24483727 10.1021/la404980g

[b21] Sundaram H. S. . One-step dip coating of zwitterionic sulfobetaine polymers on hydrophobic and hydrophilic surfaces. ACS Appl. Mater. Interfaces 6, 6664–6671 (2014). 24730392 10.1021/am500362k

[b22] Sun F. . Stealth surface modification of surface-enhanced Raman scattering substrates for sensitive and accurate detection in protein solutions. ACS Nano 9, 2668–2676 (2015). 25738888 10.1021/nn506447k

[b23] Chou Y.-N., Wen T.-C. & Chang Y. Zwitterionic surface grafting of epoxylated sulfobetaine copolymers for the development of stealth biomaterial interfaces. Acta Biomater., 10.1016/j.actbio.2016.03.046 (2016). 27045347

[b24] Gao C. . Functionalizable and ultra-low fouling zwitterionic surfaces via adhesive mussel mimetic linkages. Biomaterials 31, 1486–1492 (2010). 19962753 10.1016/j.biomaterials.2009.11.025

[b25] Zollinger H. Color chemistry: synthesis, properties and applications of organic dyes and pigments (3rd edition) 227–240 (Verlag Helvetica Chimica Acta, 2003).

[b26] He L., Li J. & Xin J. H. A novel graphene oxide-based fluorescent nanosensor for selective detection of Fe^3+^ with a wide linear concentration and its application in logic gate. Biosens. Bioelectron. 70, 69–73 (2015). 25794960 10.1016/j.bios.2015.01.075

[b27] Li R., Parvez K., Hinkel F., Feng X. & Müllen K. Bioinspired wafer-scale production of highly stretchable carbon films for transparent conductive electrodes. Angew. Chem. Int. Ed. 52, 5535–5538 (2013). 10.1002/anie.201300312 23592232

[b28] Su Y.-L. & Li C. Controlled adsorption of bovine serum albumin on poly(acrylonitrile)- based zwitterionic membranes. React. Funct. Polym. 68, 161–168 (2008).

[b29] Zhang H. & Zhu H. Preparation of Fe-doped TiO_2_ nanoparticles immobilized on polyamide fabric. Appl. Surf. Sci. 258, 10034–10041 (2012).

[b30] Joshi M., Khanna R., Shekhar R. & Jha K. Chitosan nanocoating on cotton textile substrate using layer-by-layer self-assembly technique. J. Appl. Polym. Sci. 119, 2793–2799 (2011).

[b31] Li J.-L. . Graphene oxide nanoparticles as a nonbleaching optical probe for two-photon luminescence imaging and cell therapy. Angew. Chem. Int. Ed. 51, 1830–1834 (2012). 10.1002/anie.201106102 22247035

[b32] Saif M. J., Anwar J. & Munawar M. A. A novel application of quaternary ammonium compounds as antibacterial hybrid coating on glass surfaces. Langmuir 25, 377–379 (2009). 19115872 10.1021/la802878p

[b33] Kangwansupamonkon W., Lauruengtana V., Surassmo S. & Ruktanonchai U. Antibacterial effect of apatite-coated titanium dioxide for textiles applications. Nanomed. Nanotechnol. 5, 240–249 (2009). 10.1016/j.nano.2008.09.004 19223243

[b34] Peng C., Hu W., Zhou Y., Fan C. & Huang Q. Intracellular imaging with a graphene-based fluorescent probe. Small 6, 1686–1692 (2010). 20602429 10.1002/smll.201000560

